# Hepatitis B virus X protein accelerates hepatocarcinogenesis with partner survivin through modulating miR-520b and HBXIP

**DOI:** 10.1186/1476-4598-13-128

**Published:** 2014-05-28

**Authors:** Weiying Zhang, Zhanping Lu, Guangyao Kong, Yuen Gao, Tao Wang, Qi Wang, Na Cai, Honghui Wang, Fabao Liu, Lihong Ye, Xiaodong Zhang

**Affiliations:** 1 State Key Laboratory of Medicinal Chemical Biology, Department of Cancer Research, Institute for Molecular Biology, College of Life Sciences, Nankai University, 94 Weijin Road, Tianjin 300071, P.R. China; 2 State Key Laboratory of Medicinal Chemical Biology, Department of Biochemistry, College of Life Sciences, Nankai University, Tianjin 300071, P.R. China

**Keywords:** HBx, Survivin, miR-520b, HBXIP, Hepatoma, Hepatocarcinogenesis

## Abstract

**Background:**

Hepatitis B virus X protein (HBx) plays crucial roles in hepatocarcinogenesis. However, the underlying mechanism remains elusive. We have reported that HBx is able to up-regulate survivin in hepatocellular carcinoma tissues. The oncopreotein hepatitis B X-interacting protein (HBXIP), a target of miR-520b, is involved in the development of cancer. In this study, we focus on the investigation of hepatocarcinogenesis mediated by HBx.

**Methods:**

The expression of HBx and survivin was examined in the liver tissues of HBx-Tg mice. The effect of HBx/survivin on the growth of LO2-X-S cells was determined by colony formation and transplantation in nude mice. The effect of HBx/survivin on promoter of miR-520b was determined by Western blot analysis, luciferase reporter gene assay, co-immunoprecipitation (co-IP) and chromatin immunoprecipitation (ChIP), respectively. The expression of HBx, survivin and HBXIP was detected by immunohistochemistry and real-time PCR in clinical HCC tissues, respectively. The DNA demethylation of HBXIP promoter was examined. The functional influence of miR-520b and HBXIP on proliferation of hepatoma cells was analyzed by MTT, colony formation, EdU and transplantation in nude mice *in vitro* and *in vivo*.

**Results:**

In this study, we provided evidence that HBx up-regulated survivin in the liver cancer tissues of HBx-Tg mice aged 18 M. The engineered LO2 cell lines with survivin and/or HBx were successfully established, termed LO2-X-S. MiR-520b was down-regulated in LO2-X-S cells and clinical HCC tissues. Our data revealed that HBx survivin-dependently down-regulated miR-520b through interacting with Sp1 in the cells. HBXIP was highly expressed in LO2-X-S cells, liver cancer tissues of HBx-Tg mice aged 18 M and clinical HCC tissues (75.17%, 112/149). The expression level of HBXIP was positively associated with those of HBx or survivin in clinical HCC tissues. In addition, we showed that HBx survivin-dependently up-regulated HBXIP through inducing demethylation of HBXIP promoter in LO2-X-S cells and clinical HCC tissues. In function, low level miR-520b and high level HBXIP mediated by HBx with partner survivin contributed to the growth of LO2-X-S cells *in vitro* and *in vivo*.

**Conclusion:**

HBx accelerates hepatocarcinogenesis with partner survivin through modulating tumor suppressor miR-520b and oncoprotein HBXIP.

## Background

Hepatocellular carcinoma (HCC) is one of the most malignant tumors in the world. The chronic infection of hepatitis B virus (HBV) is a crucial risk factor in the development of HCC. HBV encoded X protein (HBx) is a key player in the pathogenesis of HBV-associated liver diseases. It is able to transactivate cellular genes associated with processes such as transcription, apoptosis, signaling, and cell growth [1-3]. Our laboratory has reported that HBx plays an important role in the event, such as activating Yes-associated protein (YAP), Lin28A/B and Rab18 [4-6]. HBx transgenic (Tg) mice are able to develop hepatitis, steatosis, and dysplasia, culminating in the appearance of HCC in liver [7-9]. However, HBx alone is considered a poor transformer of human and rodent hepatic cells. In support of this, co-transfection with an oncogene, such as H-ras or myc, is necessary for accelerating hepatocarcinogenesis [10]. As an inducible factor, survivin is abundantly expressed in a hepatoma cell line harboring HBV [11]. We previously reported that HBx was able to up-regulate survivin in hepatoma cells [12]. HBx may up-regulate survivin through activation of Wnt/β-catenin signaling [13-15]. Therefore, we supposed that HBx might collaborate with survivin to accelerate hepacarcinogenesis.

Mammalian hepatitis B X-interacting protein (HBXIP) is originally identified as a binding protein of HBx [16]. Recently, it has been reported that HBXIP serves as a regulator component for the mammalian target of rapamycin (mTOR) Complex 1 activation which regulated cell growth [17]. We have reported that HBXIP acts as an oncoprotein to promote the development of breast cancer through activating some cellular genes such as S100A4, NF-κB, Interleukin-8 and c-Myc [18-20]. HBXIP up-regulates some membrane-bound complement regulatory proteins through phosphorylated extracellular signal-regulating kinase 1/2 (p-ERK1/2)/NF-κB signaling to accelerate breast tumor growth [21]. In addition, HBXIP has also been identified as an adaptor for survivin to suppress apoptosis [22]. Meanwhile, HBx may interfere with the normal function of HBXIP during prometaphase, resulting in genomic instability [23,24]. However, the function of HBXIP in the development of HCC mediated by HBx remains unclear.

Accumulating data indicated that aberrant expression of microRNAs (miRNAs) could regulate cancer development including tumorigenesis, metastasis and proliferation by serving as tumor suppressors or oncogenes. MiRNAs have essential roles in the progression of HCC and directly contribute to the development of HCC by targeting a large number of critical protein-coding genes [25,26]. Our laboratory has revealed that miR-520b targeting HBXIP and IL-8 inhibits the migration of breast cancer cells [19], which is down-regulated in breast cancer cells and sensitizes breast cancer cells to complement attack [27]. Moreover, miR-520b targeting mitogen-activated protein kinase kinase kinase 2 (MEKK2) and cyclinD1 inhibits the proliferation of liver cancer cells [28]. However, the role of miR-520b in hepatocarcinogenesis mediated by HBx remains ill-defined.

In the present study, we further investigated the role of HBx in the development of HCC. Interestingly, we identify that HBx enhances hepatocarcinogenesis with partner survivin through modulating miR-520b and HBXIP. Our finding provides new insights into the mechanism of hepatocarcinogenesis mediated by HBx.

## Results

### HBx accelerates hepatocarcinogenesis with partner survivin

**Figure 1:**
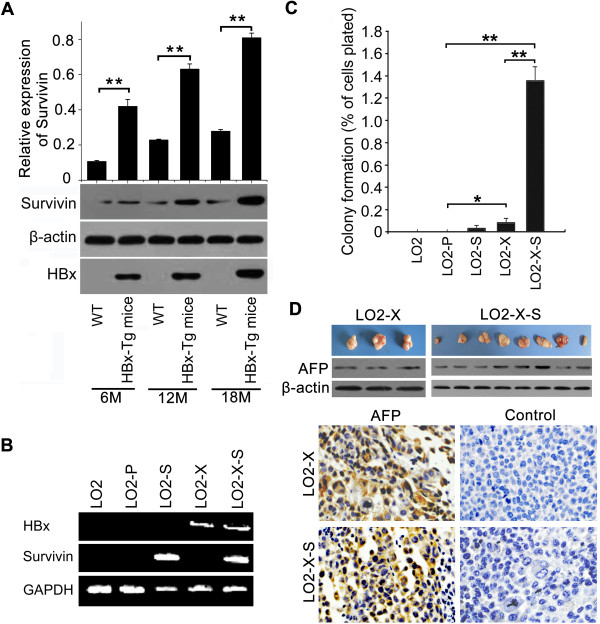
**HBx accelerates hepatocarcinogenesis with partner survivin. (A)** The expression of survivin in the liver tissues of p21-HBx Tg mice aged 6, 12 and 18 mouths versus WT littermate mice were determined by western blotting, respectively (**P < 0.01, Student’s *t* test). **(B)** The integrations of HBx and survivin genes into the genomes of LO2 cells were validated by PCR using genomic DNA as a template. GAPDH was used as a loading control. **(C)** The effect of HBx and/or survivin on cell proliferation was detected by colony-formation assay (*P < 0.05, **P < 0.01, Student’s *t* test). **(D)** Tumor formation in nude mice (n = 8 per group) injected with LO2-X or LO2-X-S cells was assessed in 3 weeks. The expression of AFP was tested in the tumor tissues from mice by western blotting and IHC analysis, respectively.

We have reported that HBx can up-regulate survivin in stable HBx transfected LO2 cells [12], however, its significance is not clear. To better understand the effect of HBx on survivin, we examined the expression levels of survivin in the liver tissues of HBx-Tg mice which were obtained from Prof. Xiao Yang [7]. Interestingly, we observed that the expression levels of survivin were increased in the liver tissues of HBx-Tg mice aged 12 M, but remarkably elevated in the liver cancer tissues of HBx-Tg mice aged 18 M (Figure 1A), supporting that HBx is capable of up-regulating survivin. Then, we speculated that survivin might be involved in the hepatocarcinogenesis mediated by HBx. To examine the role of HBx and survivin in the event, we successfully established an engineered cell line of stably HBx/survivin-transfected human immortalized liver LO2 (or mouse NIH3T3) cells (termed LO2-X-S, or 3 T3-X-S) (Figure 1B, Additional file 1: Figure S1 and Additional file 2: Table S1). Colony formation assay showed that LO2-X-S cells yielded a significantly more number of colonies relative to control cell lines (Figure 1C). We previously reported that 3 of 8 mice injected with LO2-X cells grew tumors [29]. In this study, we observed that all 8 mice injected with LO2-X-S cells formed tumors, while LO2, LO2-P and LO2-S cells failed to form any visible tumors. The expression of alpha fetoprotein (AFP, a hepatoma marker) was detectable in all tumor tissues from mice by western blotting and immunohistochemistry (IHC) (Figure 1D), suggesting that LO2 cell line is successfully transformed by HBx and survivin. Therefore, we conclude that HBx accelerates hepatocarcinogenesis with partner survivin.

### HBx down-regulates miR-520b through binding to Sp1 with partner survivin

**Figure 2:**
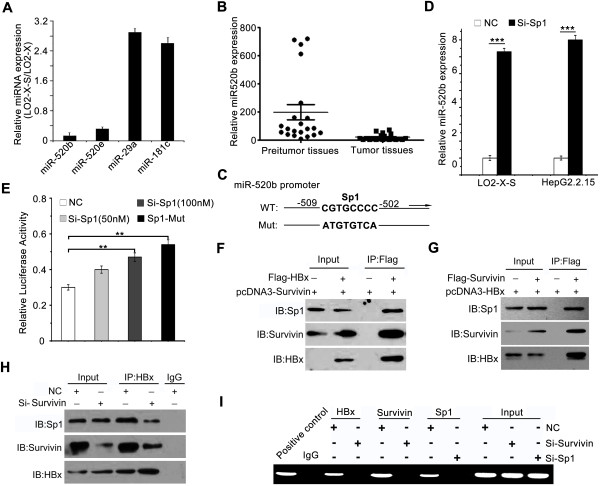
**HBx down-regulates miR-520b through binding to Sp1 with partner survivin. (A)** The expression levels of miRNA-520b, miRNA-520e, miRNA-29a and miRNA-181c were examined by qRT-PCR in LO2-X-S/LO2-X cells. **(B)** The expression of miR-520b in clinical HCC and peritumor samples was detected by qRT-PCR. **(C)** A model shows Sp1 binding site-directed mutation in the promoter region of miR-520b. **(D)** The effect of knockdown of Sp1 on miR-520b in LO2-X-S or HepG2.2.15 cells was examined by qRT-PCR analysis (***P < 0.001, Student’s *t* test). **(E)** The effect of Sp1 on miR-520b promoter in LO2-X-S cells was tested using Sp1 siRNA (Si-Sp1) or Sp1 mutant by luciferase reporter gene assays (**P < 0.01, Student’s *t* test). **(F-H)** The interaction among HBx, survivin and Sp1 in a complex was examined by co-IP. **(I)** Interaction of the complex, including HBx, survivin and Sp1, with the promoter region of miR-520b was examined by ChIP in LO2-X-S cells.

To explore the mechanism by which HBx accelerates carcinogenesis with partner survivin, we examined the expression differentiate profiles between LO2-X-S cells and LO2-X cells by miRNA microarray assay. Our data demonstrated that miR-520b and miR-520e (miR-29a and miR-181c) were remarkably down-regulated (up-regulated) (Additional file 3: Figure S2(A) and Additional file 2: Table S2). Then, we confirmed the data using qRT-PCR (Figure 2A). Moreover, we validated that the expression of miR-520b was down-regulated by qRT-PCR in LO2-X-S cells (22 HCC tissues) relative to LO2, LO2-X and LO2-S cells (their peritumor tissues) (Additional file 3: Figure S2(B) and Figure 2B). It has been reported that HBXIP which directly interacts with HBx [16] is one of the target genes of miR-520b [19]. Next, we focused on the investigation of miR-520b in the hepatocarcingenesis mediated by HBx and survivin.

We constructed the promoter of miR-520b (Additional file 3: Figure S2(C)) and searched for the possible transcription factor binding sites in miR-520b promoter using promoter analysis program TF2 SEARCH (http://www.cbrc.jp/research/db/TFSEARCH.html). We observed that the miR-520b promoter contained a transcriptional factor Sp1 binding site (Figure 2C). Furthermore, we showed that Sp1 RNAi remarkably increased the expression of miR-520b by qRT-PCR in LO2-X-S cells and HepG2.2.15 cells (Figure 2D). Luciferase reporter gene assay showed that the Sp1 siRNA removed the suppression of miR-520b in a dose-dependent manner in the cells, suggesting that Sp1 is responsible for the suppression of miR-520b expression. In addition, the Sp1 mutant of miR-520b promoter (Figure 2C) abolished the transcriptional inhibition of miR-520b in LO2-X-S cells (Figure 2E). It has been reported that HBx is able to interact with transcriptional factor Sp1 and affects its DNA binding activity [30]. Then, we examined whether survivin was involved in the interaction between HBx and Sp1 by co-immunoprecipitation (co-IP). Interestingly, we found that HBx, survivin and Sp1 formed a complex (Figure 2F-H). Chromatin immunoprecipitation (ChIP) assay further demonstrated that the miR-520b promoter gene could be detected in the anti-HBx (or anti-survivin, anti-Sp1)-immunoprecipited candidates from LO2-X-S cells, however, it was undetectable when the cells were treated with Sp1 (or survivin) siRNA (Figure 2I). Overall, we conclude that HBx down-regulates miR-520b through interacting with Sp1 with partner survivin.

### HBx up-regulates HBXIP in HBx-Tg mice and HCC tissues with partner survivin

**Figure 3:**
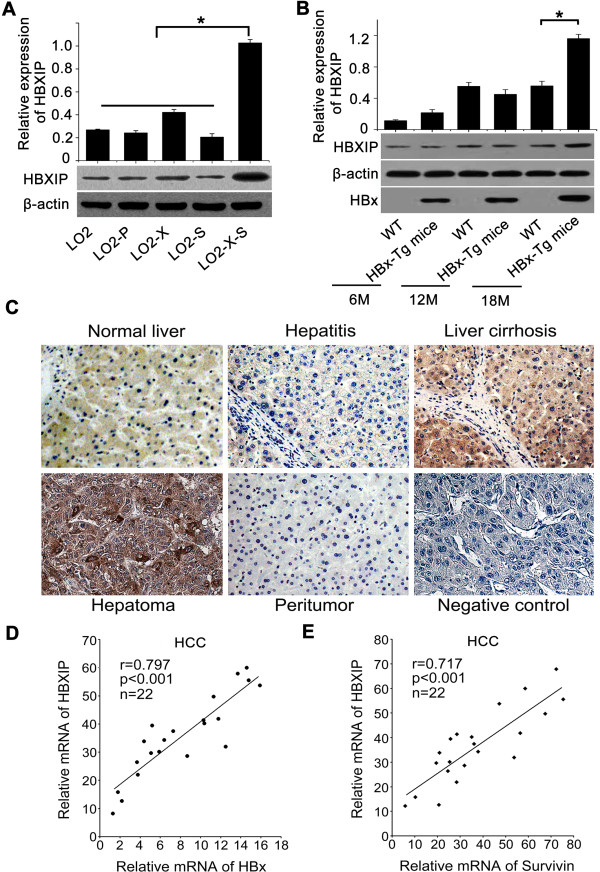
**HBx up-regulates HBXIP in HBx-Tg mice and HCC tissues with partner survivin. (A)** The expression of HBXIP was detected by western blotting in LO2 and engineered cell lines (*P < 0.05, Student’s *t* test). **(B)** The expression of HBXIP in the liver tissues of p21-HBx-Tg mice aged 6, 12 and 18 mouths versus WT littermate mice were determined by western blotting, respectively (*P < 0.05, Student’s *t* test). **(C)** Expression of HBXIP was examined by IHC staining in the clinical tissues of normal liver, hepatitis, liver cirrhosis, HCC and peritumor tissues. **(D, E)** Correlation between relative expression of HBXIP and that of HBx (or survivin) was examined by qRT-PCR in 22 cases of HCC tissues (***P <0.001, r = 0.797 or r = 0.717; Pearson’s correlation coefficient). Data presented are from three independent experiments.

We previously reported that miR-520b could target HBXIP mRNA [19]. Accordingly, we validated that in our system. Although HBx is able to directly interact with HBXIP [16], whether HBx is capable of up-regulating HBXIP remains unclear. Our data revealed that miR-520b could target HBXIP 3′UTR and reduced the expression of HBXIP at the levels of mRNA and protein in the cells (Additional file 4: Figure S3(A)), suggesting that HBx may up-regulate HBXIP with partner survivin through suppressing miR-520b. Interestingly, we observed that the expression levels of HBXIP were remarkably increased in LO2-X-S cell lines and liver cancer tissues of HBx-Tg mice aged 18 M (Figure 3A, B and Additional file 4: Figure S3(B)), suggesting that HBx accelerates carcinogenesis through up-regulating HBXIP with partner survivin. To evaluate the effect of HBV DNA on the expression of HBXIP and survivin, we transfected the pCH-9/3091 plasmid containing full-length HBV DNA into LO2 cells. Our data demonstrated that the expression levels of both HBXIP and survivin were up-regulated in the cells at the levels of mRNA and protein. Meanwhile, the expression of HBx was validated in the system (Additional file 4: Figure S3(C)). We further evaluated the effect of HBx, HBsAg and HBcAg on the expression of HBXIP and survivin in hepatoma HepG2.2.15 cells integrated HBV DNA. Interestingly, we found that the knockdown for HBx/survivin by pSi-HBx/si-survivin-1 could obviously abolish the up-regulation of HBXIP. But, si-HBs (for HBsAg mRNA) and si-HBc (for HBcAg mRNA) failed to work (Additional file 4: Figure S3(D)), suggesting that HBx and survivin (not HBsAg and HBcAg) are responsible for the up-regulation of HBXIP in the cells [31].

Previously, we reported that the positive rates of HBxAg and survivin in HCC tissues were 76.5% and 88.2%, respectively [12]. In this study, we further examined the expression of HBXIP in clinical HCC tissues. IHC showed that the expression of HBXIP was positive in 112 out of 149 (75.17%) cases of HCC tissues, of which 59 out of 112 (52.68%) tissues exhibited stronger HBXIP staining (Figure 3C and Table 1). In contrast, 20 peritumor samples, 30 normal liver samples and 10 hepatitis samples were weak staining for HBXIP. Furthermore, we found that the up-regulation of HBXIP was significantly correlated with those of HBx (or survivin) in 22 human HCC tissues by quantitative real-time polymerase chain reaction (qRT-PCR) (r = 0.797 or 0.717, *p* <0.001, Pearson′s correlation, Figure 3D, E). Thus, our data suggest that HBXIP is up-regulated in HCC tissues with partner survivin.

**Table 1:** The Expression of HBXIP in human liver tissues

**Tissues**	**Total (No.)**	**Positive (+, No.)**	**Positive (++ to +++, No.)**	**Strongly Positive rate (%)**
Normal liver	10	10	0	0.00
Hepatitis	10	10	0	0.00
Liver cirrhosis	40	38	2	5.00
Hepatocarcinoma	149	37	112	75.17*
Peritumor	20	20	0	0.00

Expression: positive, + to +++. X^2^ test, ^*^P < 0.01 (versus Liver cirrhosis).

### HBx survivin-dependently up-regulates HBXIP *via* DNA demethylation of HBXIP

**Figure 4:**
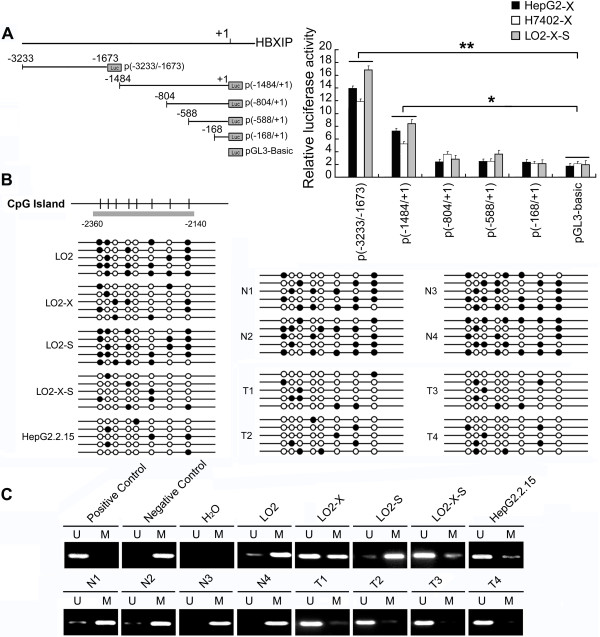
**HBx survivin-dependently up-regulates HBXIP *via *DNA demethylation of HBXIP. (A)** Schematic representation of the HBXIP promoter was shown. The activities of different fragments from HBXIP promoter were tested by luciferase reporter gene assays in HepG2-X, H7402-X and LO2-X-S cells (*P < 0.05, **P < 0.01, Student’s *t* test). **(B, C)** The methylation levels of HBXIP CpG sites were examined by bisulfite sequencing and MSP analysis in the cells and clinical tissues (n = 4), respectively. Eight CpG sites were analyzed. Closed and open circle present methylated and unmethylated CpG site, respectively. N represents adjacent nontumorous tissue; T represents HCC tissue. Bands in the ‘M’ and ‘U’ lanes are PCR products obtained with methylation-specific and unmethylation-specific primers, respectively.

In this study, we demonstrated that HBx up-regulated HBXIP with partner survivin through down-regulating miR-520b targeting HBXIP mRNA. Next, to explore the other mechanism of up-regulating HBXIP by HBx, we examined the effect of HBx and suvivin on DNA methylation of HBXIP. We cloned and identified the core region of HBXIP promoter. Various fragments of HBXIP 5'-flanking region, including −3233/-1673, −1484/+1, −804/+1, −588/+1, and −168/+1, were cloned and transiently transfected into HepG-X (or H7402-X, LO2-X-S) cells, respectively. As shown in Figure 4A, p(−3233/-1673) displayed the highest promoter activities among them. Interestingly, we observed a typical CpG island in the region of −2360 ~ −2140 by using CpG Island Searcher (http://cpgislands.usc.edu/) or MethylPrimer Express Software v1.0. Bisulfite sequencing analysis and methylation-specific PCR (MSP) showed that the CpG sites were demethylated in LO2-X-S/HepG2.2.15 cells and clinical HCC tissues (n = 4). In contrast, the CpG sites were highly methylated in LO2, LO2-S cells and nontumorous liver tissues (Figure 4B, C). It suggests that HBx up-regulate HBXIP in hepatoma cells through inducing demethylation of CpG islands of HBXIP promoter with partner survivin.

### MiR-520b/HBXIP modulates proliferation of LO2-X-S cells *in vitro*

**Figure 5:**
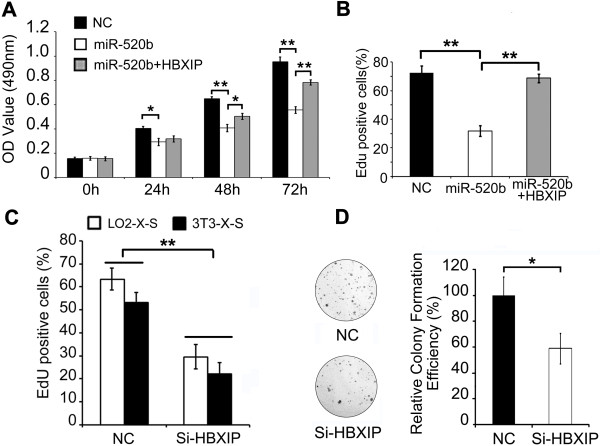
**MiR-520b/HBXIP modulates proliferation of LO2-X-S cells *in vitro*. (A-D)** The effects of miR-520b, miR-520b/HBXIP or si-HBXIP on proliferation of LO2-X-S cells were examined by MTT, EdU incorporation or colony-formation assays, respectively. Si-NC refers to negative control of HBXIP siRNA. Data are shown as mean ± SD of three independent experiments (*P < 0.05, **P < 0.01, Student’s *t* test).

Next, we evaluated the function of miR-520b/HBXIP mediated by HBx with partner survivin in carcinogenesis. MTT and EdU incorporation assays demonstrated that overexpression of miR-520b resulted in the inhibition of proliferation of LO2-X-S cells, while the introduction of HBXIP significantly rescued the suppression by miR-520b (Figure 5A, B). Moreover, the HBXIP siRNA inhibited the proliferation of LO2-X-S (or 3 T3-X-S) cells by EdU and colony-formation assays (Figure 5C, D). It suggests that HBx accelerates proliferation of LO2-X-S cells *in vitro* through down-regulating miR-520b and up-regulating HBXIP.

### MiR-520b/HBXIP modulates growth of LO2-X-S cells *in vivo*

**Figure 6:**
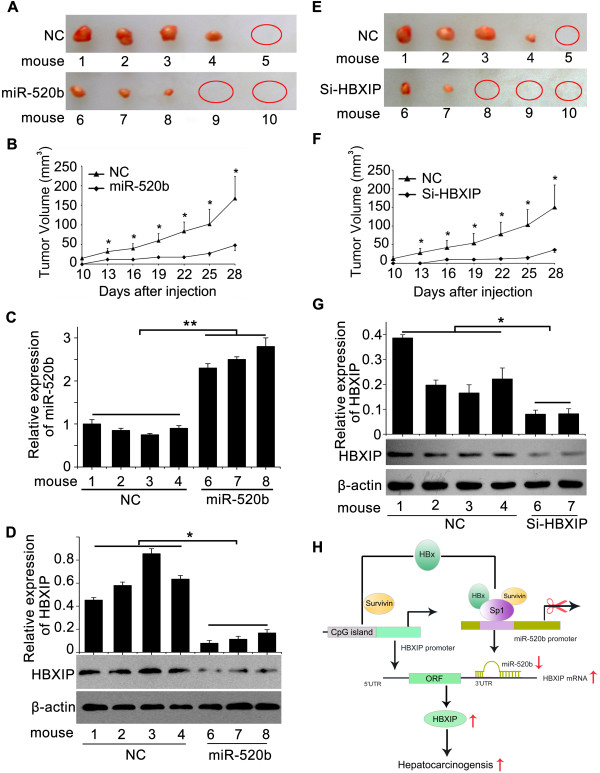
**MiR-520b/HBXIP modulates growth of LO2-X-S cells *in vivo. *(A-G)** The effect of miR-520b or si-HBXIP on the growth of LO2-X-S cells was detected by xenograft assay. The expression levels of miR-520b and HBXIP in the tumor tissues from mice (n = 5, each group) were determined by qRT-PCR or western blot analysis, respectively. Data are shown as mean ± SD of three independent experiments (*P < 0.05, **P < 0.01, Student’s *t* test). **(H)** A model of HBx accelerating hepatocarcinogenesis with partner survivin.

We further examined the role of miR-520b/HBXIP in tumor growth *in vivo*. We previously reported that 3 of 8 nude mice injected with LO2-X cells grew tumors [29]. In this study, we observed that all 8 nude mice injected with LO2-X-S cells formed tumors, while LO2, LO2-P and LO2-S cells failed to form any visible tumors. Then, we observed that the introduction of miR-520b into LO2-X-S cells led to a significant reduction of tumor formation (n = 5, each group). Meanwhile, the expression levels of miR-520b by qRT-PCR in the tumor tissues from mice (Figure 6A-C), suggesting that the levels of miR-520b are correspondence to the growth ability. Interestingly, the expression levels of HBXIP were decreased in the system by western blotting (Figure 6D), suggesting that the inhibition of HBXIP contributes to the suppression of tumor growth in the mice. Further data validated that the inhibition of HBXIP by siRNA obviously resulted in the reduction of tumor formation and depressed the growth of LO2-X-S cells in mice (n = 5, each group), meanwhile, the expression levels of HBXIP in the tumor tissues from mice were correspondence to the growth of tumor by western blotting (Figure 6E-G). Therefore, we conclude that miR-520b suppresses and HBXIP accelerates LO2-X-S cells growth *in vivo*.

## Discussion

It has been reported that HBx is a key factor in hepatocarcinogenesis [32]. The co-transfection with an oncogene, such as H-ras or myc, is necessary for accelerating hepatocarcinogenesis [10]. HBx can promote initiation and progression of hepatocellular carcinoma through cooperating with Kras involved in Ras pathway [33]. We previously reported that HBx was able to up-regulate survivin in hepatoma cells [12]. Therefore, we are interested in whether HBx can accelerate hepatocarcinogenesis through cooperating with survivin.

Interestingly, in this study we validated that survivin was up-regulated in the liver tissues from HBx-Tg mice in a time course manner. To demonstrate the significance of survivin up-regulation in HBx-induced hepatocarcinogenesis, we successfully generated an engineered cell line model (termed LO2-X-S) that LO2 cells stable transfected with both HBx and survivin. We previously reported that 3 of 8 mice injected with LO2-X cells stably transfected HBx grew tumors [29]. Strikingly, in this study we observed that all 8 mice injected with LO2-X-S cells formed tumors. It implies that survivin is an important partner of HBx in tumorigenesis.

Next, to better understand the underlying mechanism of tumorigenesis mediated by HBx with partner survivin, we assessed the miRNA differentiate expression profiles between LO2-X-S cells and LO2-X cells using miRNA microarray assay. Our data showed that miR-520b was down-regulated in LO2-X-S cells, relative to LO2-X cells. We previously reported that HBXIP was a target gene of miR-520b in breast cancer cells [19]. Therefore, we concerned that miR-520b targeting HBXIP might be involved in the HBx-induced hepatocarcinogenesis. Moreover, we identified that HBx was able to form a complex with survivin and transcription factor Sp1, resulting in the down-regulation of miR-520b through inactivating miR-520b promoter. Consistent with our previous reports [19,27,28], our finding suggests that miR-520b is a novel tumor suppressor gene.

Then, we moved to the investigation of HBXIP in HBx-induced hepatocarcinogenessis. It has been reported that HBXIP functions as a cofactor of survivin in apoptosis suppression [16,22]. HBXIP is a critical target of viral HBx for promoting genetic instability through formation of defective spindles and subsequent aberrant chromosome segregation [23]. HBXIP is a regulator of centrosome duplication, required for bipolar spindle formation in HeLa human carcinoma cells and primary mouse embryonic fibroblast cells [24]. Our laboratory also shows that HBXIP is a novel oncoprotein in breast cancer [18,19]. In this study, we used the model of HBx-Tg mice to evaluate expression of survivin and HBXIP in HBx-induced hepatocarcinogenesis. Strikingly, we observed that both of survivin and HBXIP were markedly up-regulated in HBx-Tg mice, however, the up-regulation of survivin was earlier (at 12 M mice) than that of HBXIP (at 18 M mice). Moreover, the expression of HBXIP stayed lower in the liver at 12 M HBx-Tg mice which didn’t develop HCC. However, the HBXIP was highly expressed in the liver cancer at 18 M HBx-Tg mice that developed HCC. Therefore, it strongly suggests that HBx first up-regulates survivin and then increases the expression of HBXIP through collaborating with survivin. In clinical, we found that 20 peritumor samples, 30 normal liver samples and 10 hepatitis samples were weak staining for HBXIP. But, HBXIP was highly expressed in HCC tissues, positively associating with the expression levels of HBx and survivin. The above data are consistent with the data in HBX-Tg mice.

It has been reported that the global patterns of DNA methylation are altered in many cancers, including HCC [34-36]. To explore the mechanism by HBx and survivin up-regulate HBXIP, we observed the promoter region of HBXIP using epigenetics analysis. Interestingly, we found that there were CpG islands in the promoter region. Thus, we supposed that the methylation regulation might be involved in the up-regulation of HBXIP mediated by HBx and survivin. Accumulating data reported that HBx had an effect on DNA methyltransferase [37,38]. However, the role of survivin in methylation regulation has not been reported. Surprisingly, we showed that HBx and survivin resulted in demethylation of HBXIP promoter in the cells. The methylation modification of HBXIP was observed in the cell lines and clinical liver cancer tissues. Therefore, it suggests that HBx up-regulates HBXIP with partner survivin not only through down-regulating miR-520b targeting HBXIP mRNA but also inducing demethylation of HBXIP promoter. The possible mechanism may be related to that the overexpression of both HBx and survivin suppresses the methylase (or methyltransferase), or activate demethylase in the cells. The mechanism needs to be further investigated in detail. Recently, our laboratory has reported that the oncoprotein HBXIP serves as co-activator of transcriptional factors in breast cancer. The nuclear import of oncoprotein HBXIP depends on interacting with c-Fos and phosphorylation of both proteins in breast cancer cells [39-41]. Thus, our finding suggests that HBXIP as a crucial oncoprtein contributes to the HBx-induced hepatocarcinogenesis. In function, we found that miR-520b and HBXIP were responsible for the growth of LO2-X-S cells *in vitro* and *in vivo*. Therapeutically, all the factors, such as HBx, survivin and HBXIP, may serve as targets in HBV-associated HCC.

## Conclusion

We summarize a model that HBx accelerates hepatocarcinogenesis with partner survivin (Figure 6H). On the one hand, HBx survivin-dependently down-regulates tumor suppressor miR-520b through modulating transcription factor Sp1 and then miR-520b inhibits the expression of oncoprotein HBXIP through targeting its 3'UTR; on the other hand, HBx induces demethylation of HBXIP promoter with partner survivin, resulting in up-regulation of HBXIP. Our finding provides new insights into the mechanism by which HBx accelerates hepatocarcinogenesis.

## Methods

### Plasmid constructs

The plasmid pCMV-X was used as a template for subcloning HBx into the pCMV-Tag2B vector (termed Flag-X) [12]. One 3′-deleted DNA fragment and four 5′-deleted DNA fragments of various sizes were amplified by PCR from the 5′-flanking region of HBXIP. To generate a series of reporter constructs (p-3233/-1673, p-1484/+1, p-804/+1, p-588/+1 and p-168/+1), each fragment was inserted between the Kpn I and Hind III sites in the pGL3-basic vector (Promega, USA). To clone the miR-520b promoter into the pGL3-basic vector, a 1 kb section of the putative promoter region (from −1 to −1000) was amplified by PCR and cloned into the pGL3-basic vector. The transcription factor binding site mutants of the miR-520b promoter were cloned using the Fast Mutagenesis System (TransGen Biotech, China). All primers used are listed in Additional file 2: Table S2.

### Cell culture and generation of stable cell lines

The human immortalized liver LO2, HepG2, HepG2.2.15 and NIHT3T cell lines were purchased from Nanjing KeyGEN Biotech Co., Ltd (Nanjing, China). The co-transfection with the plasmids of pCMV-X and pcDNA3-sur [12] was performed in LO2 cells using Lipofectamine 2000 (Invitrogen, USA) as described [12]. After 48 hours, the transfected cells were incubated in selection medium containing 500 μg/ml or 800 μg/ml G418 (Genview, USA). The transfection efficiencies were tested by PCR (one primer is from survivin and another is from the vector), western blotting, as indicated. Then, the engineered cell line was termed LO2-X-S. All the engineered cell lines were cultured in RPMI Medium 1640 (GIBCO, USA) containing 100 U/ml penicillin, 100 μg/ml streptomycin and 10% fetal calf serum (FCS). HepG2 cells, HepG2-X cells [42] and HepG2.2.15 cells were cultured in Dulbecco’s Modified Eagle’s medium (DMEM, GIBCO) containing 100 U/ml penicillin, 100 μg/ml streptomycin and 10% FCS.

### Patient samples

The 22 paired HBV-related HCC tissues and the corresponding nearby noncancerous livers used in this study were obtained from patients who underwent radical resection at Tianjin First Center Hospital (Tianjin, China). Specimens were immediately frozen and stored at −80°C. Clinicopathological information about patients was obtained from patient records and was summarized in Table 1. All patients were diagnosed with primary HCC, and none had received previous radiotherapy or chemotherapy before surgery. Informed consent was obtained from each patient and the study was approved by the Institute Research Ethics Committee at Nankai University.

### MiRNA microarray

MiRNA microarrays were performed by CapitalBio (CapitalBio Corp, China) as described in detail on the CapitalBio website (http://www.capitalbio.com). The miRNAs that showed >2-fold or <0.5 changes in expression were considered to have changed significantly and are shown in (Additional file 2: Table S2).

### RNA extraction, reverse-transcription and quantitative real-time polymerase chain reaction (qRT-PCR)

Total RNA of cells (or liver tissues) were extracted using TRIzol reagent (Invitrogen) and qRT-PCR was performed according to the manufacturer’s instructions. The expression of specific genes or miRNAs was tested by the comparative Ct method using 2^-ΔΔCt^[43]. Primers used are listed in (Additional file 2: Table S2). The HBV DNA in the supernatants of LO2 cells transfected with PCH9/3091 containing a genome length HBV sequence was extracted with a commercial kit (QIAGEN, USA) [44]. HBV DNA copies were quantified by qRT-PCR according to a diagnostic kit for quantification of HBV DNA (Da An Gene, China).

### Western blotting

Western blotting was carried out in the liver of HBx-Tg mice, cell lines and tumor tissues from mice using standard protocols [29]. The following primary antibodies were used: actin (NeoMarkers, USA), HBXIP, [45] HBx, [46] HBs (Zhongshan-Golden Bridge, ZM-0122, China), HBc (Zhongshan-Golden Bridge, ZM-0421, China), Sp1 (McAb, Epitomics, ab77441, USA), survivin (PcAb, Labvision, PA1-16836, USA), Flag (PcAb, Abcam, ab93713, UK) and α-fetoprotein ( PcAb, NeoMarkers, PA5-11480, USA). The data were analyzed by Image-Pro Plus 6.0 software.

### RNA interference (RNAi) and miRNA

The plasmid of pSilencer3.0-X encoding HBx mRNA was used in transfection of HepG2.2.15 cells as described above [12]. The short interfering RNA (siRNA) duplexes targeting survivin, HBXIP [18,47] were synthesized by Ribobio Co. Lit. (Guangzhou, China) as previously described. MiR-520b mimics and its inhibitor (anti-miR-520b) for *in vitro* transfection, and their respective negative controls were from Ribobio Co. Lit. The sequences of miR-520b mimics and its inhibitor are 5' AAAGUGCUUCCUUUUAGAGGG 3' and 5' CCCUCUAAAAGGAAGCACUUU 3'. The transfected cells were lysed after 48 hours. Western blotting or RT-PCR was used to determine the expression levels of survivin and HBXIP.

### Luciferase reporter gene assay

H7402-X, HepG2-X cells or LO2 and various LO2-engineered cell lines were plated in 24-well plates (3× 10^4^ cells/well). The cells were transfected with plasmids using Lipofectamine 2000 (Invitrogen). At 48 hours post-transfection, a standard dual luciferase reporter assay was performed, and the results were normalized using a co-transfected pRL-TK plasmid containing the Renilla luciferase gene (Promega). All experiments were performed at least three times.

### Co-immunoprecipitation assay

Immunoprecipitations were performed using anti-flag M2 agarose (Sigma,USA) according to the manufacturer’s instructions. The immunoprecipitated proteins were then identified by western blotting as described above using the anti-HBx antibody, anti-survivin antibody, anti-Sp1 and anti-HBXIP antibody.

### Chromatin immunoprecipitation assay (ChIP)

The ChIP assay was performed using the EpiQuikTM Chromatin Immunoprecipitation Kit from Epigentek Group Inc. (Brooklyn, NY). Protein–DNA complexes were immunoprecipitated with HBx, survivin or Sp1, with Anti-RNA polymerase II as a positive control antibody and normal mouse IgG as a negative control antibody. PCR amplification was performed using 1 μl of each DNA sample. Amplification of soluble chromatin prior to immunoprecipitation was used as an input control.

### Immunohistochemistry analysis

The normal human liver, hepatitis, liver cirrhosis and hepatocellular carcinoma tissue microarrays were obtained from the Xi'an Aomei Biotechnology Co., Ltd. (Xi'an, China). Immunohistochemical staining of samples was performed as previously reported [12]. The percentage of cells showing positive nuclear and/or cytoplasmic staining for HBXIP was calculated by reviewing the entire slide. On the basis of the percentage of cells with positive nuclear and/or cytoplasmic staining, staining patterns were classified on a four-grade scale: 0, <10% cells with positive nuclear and/or cytoplasmic staining; 1+, 10–30% positive cells; 2+, 30–50% positive cells; 3+ >50% positive cells. Categorization of immunostaining intensity was performed by two or three independent observers. Light microscopic images were documented using a Nikon Eclipse Ti-U fluorescence microscope (Nikon Corp) with an attached SPOT RT digital camera (Diagnostic Instruments, Inc.).

### Gene-specific DNA methylation

Genomic DNA was extracted from the cells (or liver tissues) using a TIANamp Genomic DNA Kit (TIANGEN BIOTECH CO.) according to the manufacturer’s instructions. The methylation statuses of various genes were determined by bisulfite sequencing PCR after sodium bisulfite conversion of the DNA using an EZ DNA Methylation-GoldTM Kit (Zymo Research, USA) according to the manufacturer’s instructions. This method is based on the differential sensitivities of methylated and unmethylated CpG to bisulfite treatment. The modified DNA was then used as a PCR template to amplify either unmethylated or methylated bisulfite-converted complementary sequences of the promoter of interest. The primers were designed using MethPrimer software (http://www.urogene.org/methprimer/). The primer sequences are given in (Additional file 2: Table S2). PCR products were gel-extracted and subcloned into pMD18-T vectors (TAKARA, China), and five clones from each sample were sequenced for DNA sequencing.

### Analysis of cell proliferation

For cell proliferation assays, LO2 or various engineered cells were seeded in 96-well plates (1000 cells/well) for 24 hours before transfection and 3-(4,5-dimethylthiazol-2-yl) -2,5-diphenyltetrazolium bromide (MTT) (Sigma) assays were used to assess cell proliferation every day from the first day until the third day after transfection. The protocol was described previously [48]. 5-ethynyl-2’-deoxyuridine (EdU) incorporation assay was carried out using the Cell-Light TM EdU imaging detecting kit according to the manufacturer’s instructions (RiboBio, China).

### Colony formation assay

Anchorage-independent colony formation by these cell lines was determined as described previously [49], with some modifications. For colony formation analysis, LO2 and various engineered cells were seeded in six-well plates. Cells were transfected with RNA interferences targeting HBXIP using Lipofectamine 2000. Cells were grown for 9 to 14 days. Once colonies were visible, they were stained with methylene blue and photographed. All assays were repeated at least three times. The colonies were counted using a dissecting microscope.

### Transplantation in nude mice

The tumorigenicity of LO2 or the LO2 engineered cell lines were measured as follows. Cells were harvested by trypsinization, washed twice with sterile phosphate-buffered saline, and resuspended at a concentration of 1 × 10^7^ cells per ml. Aliquots of 0.1-0.2 ml were injected subcutaneously into 4- to 6-week-old Balb/c athymic nude mice. Mice were observed at periodic intervals, and photographs were taken 3–4 weeks after the injection. Tumor volume (V) was monitored by measuring the length (L) and width (W) with calipers and calculated with the formula (L × W^2^) × 0.5.

### Statistical analysis

All values are presented as means ± SD. Each value is the mean of at least three separate experiments in each group. Data were analyzed to compare two groups using a Student’s *t* test. *P* values of less than 0.05 were considered to be statistically significant. All statistical analysis was performed using SPSS13.0 software (Chicago, IL).

## Abbreviations

AFP: Alpha-fetoprotein; ChIP: Chromatin immunoprecipitation; co-IP: Co-immunoprecipitation; CREB: cAMP response element-binding protein; EdU: Ethynyldeoxyuridine; EMSA: Electrophoretic mobility shift assay; HBcAg: Hepatitis B core antigen; HBsAg: Hepatitis B surface antigen; HBV: Hepatitis B virus; HBx: Hepatitis B virus X protein; HBXIP: Hepatitis B X-interacting protein; HCC: Hepatocellular carcinoma; mTOR: Mammalian target of rapamycin; IHC: Immunohistochemistry; IL-8: Interleukin-8; M: Month; MEKK2: Mitogen-activated protein kinase kinase kinase 2; miRNA: microRNA; mut: Mutant; mRNA: Messenger RNA; MTT: 3-(4,5)-dimethylthiahiazo(−z-y1)-3,5-di-phenytetrazoliumromide; NF-κB: Nuclear factor kappa light-chain enhancer of activated B cells; qRT-PCR: Quantitative real-time polymerase chain reaction; RNAi: RNA interference; siRNA: Small interfering RNA; Sp1: Specificity protein 1; S100A4: S100 calcium-binding protein A4; Tg: Transgenic; 3' UTR: 3'untranslated region; wt: Wild type; YAP: Yes-associated protein.

## Competing interests

The authors declared that they have no competing interests.

## Authors’ contributions

XZ and LY designed the experiments, supervised the project and wrote the manuscripts. WZ and GK designed and performed the experiments, analyzed and interpreted data, and wrote the manuscripts; YG, ZL, TW, and QL performed the experiments, analyzed and interpreted data; QW, NC and HW analyzed and interpreted data. All authors read and approved the final manuscript.

## Supplementary Material

Additional file 1: Figure S1 The engineered cell lines are established. The integrations of HBx and survivin genes into the genomes of NIH3T3 cells were validated by PCR using genomic DNA as a template. GAPDH was used as a loading control. Click here for file

Additional file 2: Table S1 List of primers used in this paper. **Table S2.** MicroRNAs are regulated in LO2-X-S cells. All differentially expressed miRNAs have a q value < 0.01(false-positive rate). *P < 0.05, Student’s *t* test. MiRNAs marked # were further tested by qRT-PCR. Click here for file

Additional file 3: Figure S2 HBx down-regulates miR-520b through Sp1 with partner survivin. (A) The miRNA expression profiles were determined by miRNA microarray in the cells. The image shows signals from probes hybridized onto miRNA chips (up panel). The scatter plot graph of Cy3-1abeled and Cy5-1abeled probes hybridized with the microarray was showed (down panel). Each point on the plot represents a miRNA hybridization signal. (B) The expression level of survivin was detected in LO2, LO2-X, LO2-S and LO2-X-S cells (*P < 0.05, Student’s *t* test). (C) The activity of the miR-520b promoter was analyzed by luciferase reporter gene assay in LO2-X cells. (D) The interference efficiency of two siRNAs targeting survivin (si-survivin-1 and si-survivin-2) was detected in LO2-X-S cells by western blotting (*P < 0.05, Student’s *t* test). Click here for file

Additional file 4: Figure S3 HBx up-regulates HBXIP in HBx-Tg mice and HCC tissues with partner survivin. (A) The effect of miR-520b and pre-miR-520b on the activities of HBXIP 3′UTR was analyzed at the levels of promoter, mRNA and protein in LO2-X-S cells by luciferase reporter gene assay, RT-PCR and western blotting, respectively (*P < 0.05, Student’s *t* test). (B) The expression of HBXIP was detected by western blotting in the engineered NIH3T3 cell lines. (C) The expression levels of survivin and HBXIP (or HBx) in LO2 cells transfected with pCH-9/3091 was detected by qRT-PCR and western blotting, respectively (*P < 0.05, Student’s *t* test). (D) The effect of HBx and/or survivin, HBs, HBc on the expression of HBXIP and survivin was examined by western blotting in HepG2.2.15 cells, respectively. Meanwhile, the interference efficiency of HBx, survivin, HBsAg (HBs) and HBcAg (HBc) was examined by western blotting in the cells. Click here for file
