# Supplementary figures and images for: Hepatitis B virus X protein accelerates hepatocarcinogenesis with partner survivin through modulating miR-520b and HBXIP
Source: Mol Cancer. 2014 May 28;13:128. doi: 10.1186/1476-4598-13-128 (PMC4046021; doi:10.1186/1476-4598-13-128)

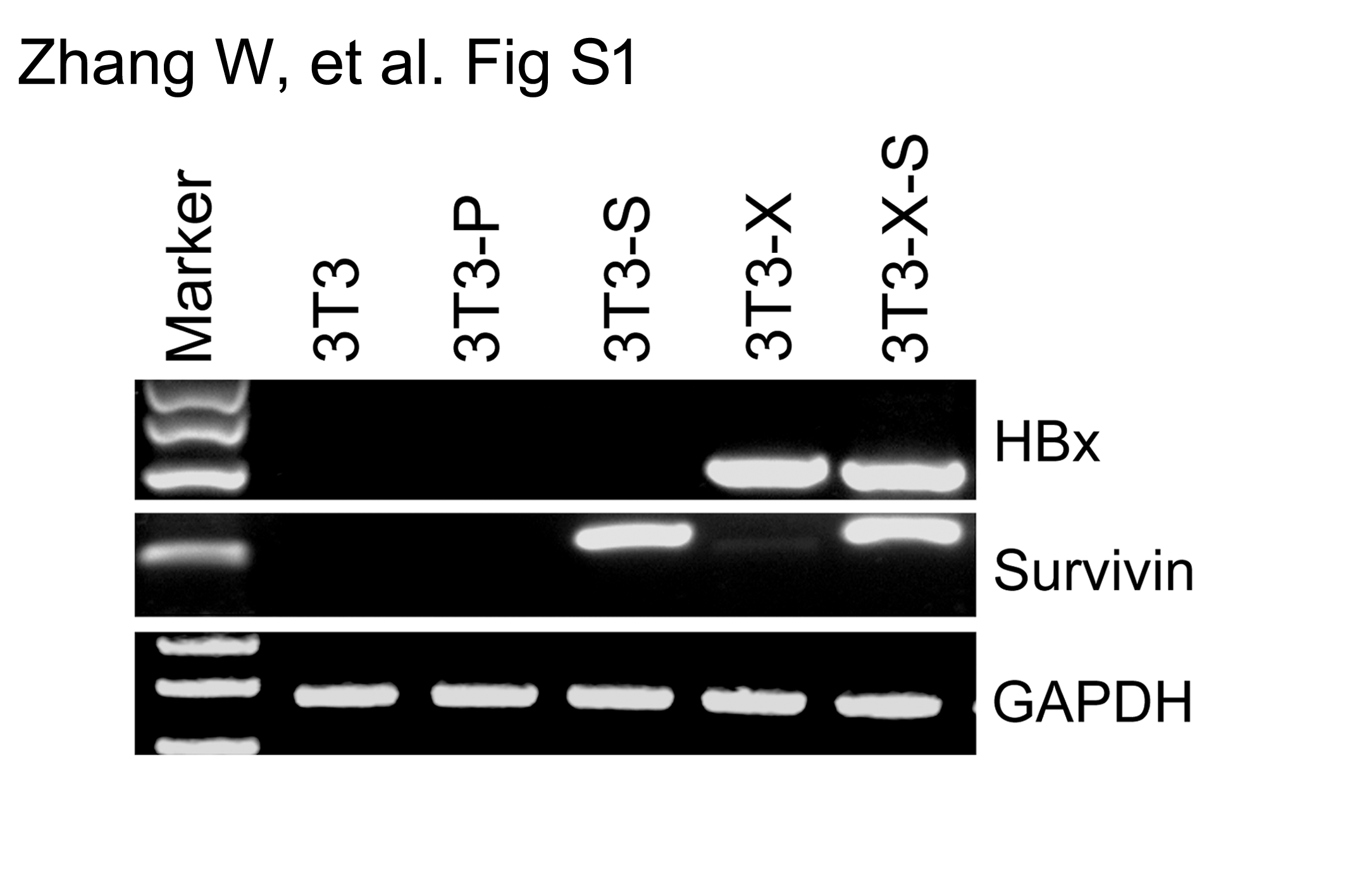

Supplement: Additional file 1: Figure S1 — The engineered cell lines are established. The integrations of HBx and survivin genes into the genomes of NIH3T3 cells were validated by PCR using genomic DNA as a template. GAPDH was used as a loading control. [file 1476-4598-13-128-S1.tiff]

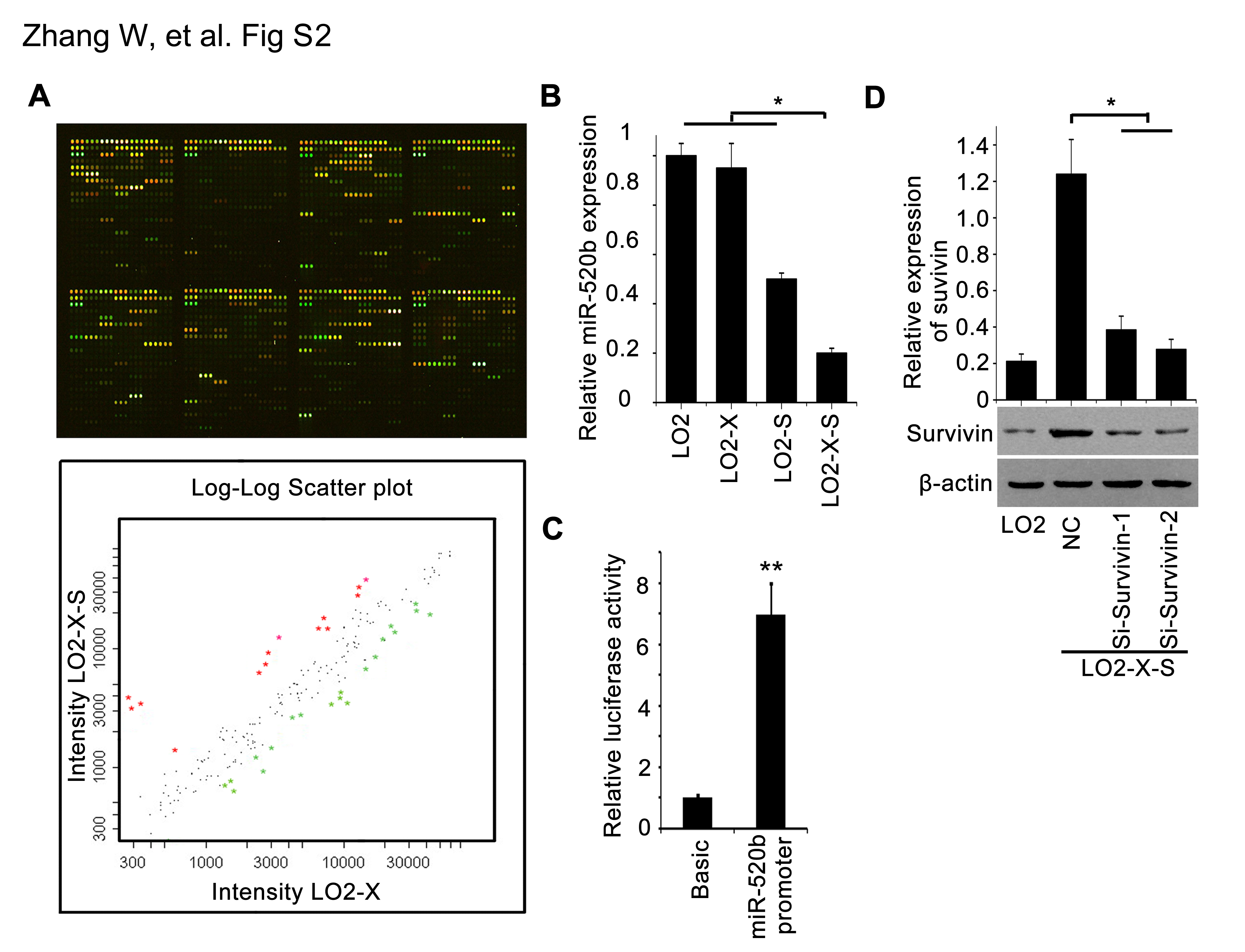

Supplement: Additional file 3: Figure S2 — HBx down-regulates miR-520b through Sp1 with partner survivin. (A) The miRNA expression profiles were determined by miRNA microarray in the cells. The image shows signals from probes hybridized onto miRNA chips (up panel). The scatter plot graph of Cy3-1abeled and Cy5-1abeled probes hybridized with the microarray was showed (down panel). Each point on the plot represents a miRNA hybridization signal. (B) The expression level of survivin was detected in LO2, LO2-X, LO2-S and LO2-X-S cells (*P < 0.05, Student’s t test). (C) The activity of the miR-520b promoter was analyzed by luciferase reporter gene assay in LO2-X cells. (D) The interference efficiency of two siRNAs targeting survivin (si-survivin-1 and si-survivin-2) was detected in LO2-X-S cells by western blotting (*P < 0.05, Student’s t test). [file 1476-4598-13-128-S3.tiff]

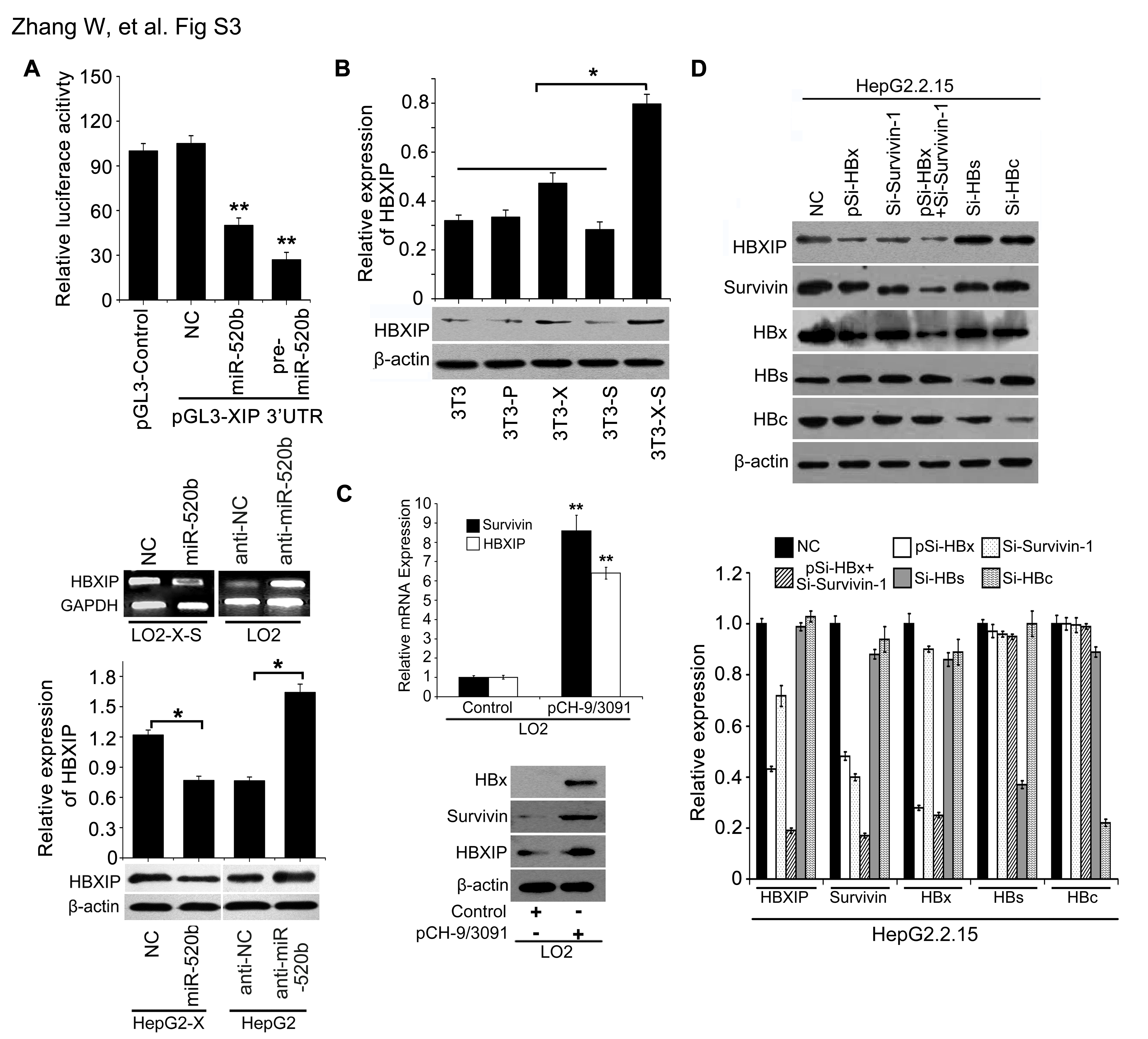

Supplement: Additional file 4: Figure S3 — HBx up-regulates HBXIP in HBx-Tg mice and HCC tissues with partner survivin. (A) The effect of miR-520b and pre-miR-520b on the activities of HBXIP 3′UTR was analyzed at the levels of promoter, mRNA and protein in LO2-X-S cells by luciferase reporter gene assay, RT-PCR and western blotting, respectively (*P < 0.05, Student’s t test). (B) The expression of HBXIP was detected by western blotting in the engineered NIH3T3 cell lines. (C) The expression levels of survivin and HBXIP (or HBx) in LO2 cells transfected with pCH-9/3091 was detected by qRT-PCR and western blotting, respectively (*P < 0.05, Student’s t test). (D) The effect of HBx and/or survivin, HBs, HBc on the expression of HBXIP and survivin was examined by western blotting in HepG2.2.15 cells, respectively. Meanwhile, the interference efficiency of HBx, survivin, HBsAg (HBs) and HBcAg (HBc) was examined by western blotting in the cells. [file 1476-4598-13-128-S4.tiff]
