# Supplementary material for: Hepatitis B virus X protein accelerates hepatocarcinogenesis with partner survivin through modulating miR-520b and HBXIP
Source: Mol Cancer. 2014 May 28;13:128. doi: 10.1186/1476-4598-13-128 (PMC4046021; doi:10.1186/1476-4598-13-128)
Supplement: Additional file 2: Table S1 — List of primers used in this paper. Table S2. MicroRNAs are regulated in LO2-X-S cells. All differentially expressed miRNAs have a q value < 0.01(false-positive rate). *P < 0.05, Student’s t test. MiRNAs marked # were further tested by qRT-PCR. [file 1476-4598-13-128-S2.doc]

**Hepatitis B virus X protein accelerates hepatocarcinogenesis with partner survivin through modulating miR-520b and HBXIP**

Weiying Zhang^1^, Zhanping Lu^1^, Guangyao Kong^1^, Yuen Gao^1^, Tao Wang^1^, Qi Wang^1^, Na Cai^1^, Honghui Wang^2^, Fabao Liu^2^, Lihong Ye^2^ and Xiaodong Zhang^1*^

**Additional file 2: Table S1.** List of primers used in this paper

| **Gene** | **Primer** | **Sequence (5'-3')** |
| --- | --- | --- |
| **Primers for RT-PCR and Real-Time PCR** | | |
| HBx | forward | GGCTCGAGATGGCTGCTAGGCTGTGC |
|  | reverse | GGCGAATTCAGAAGTCGTCGTCGTCC |
| Survivin | forward | TGCAGACCAGGTCTGGGCATG |
|  | reverse | GAAAGCGCAACCGGACGAAT |
| HBXIP | forward | GACGAATTCATGGAGGCGACCTTGGAGCA |
|  | reverse | GATCTCGAGTCAAGAGGCCATTTTGTGCA |
| GAPDH | forward | GGTCATCCCTGAGCTGAACG |
|  | reverse | TCCGTTGTCATACCAGGAAAT |
|  | forward | CATCACCATCTTCCAGGAGCG |
|  | reverse | TGACCTTGCCCACAGCCTTG |
| MiR-520b | forward | AAAGTGCTTCCTTTTAGAGGG |
|  | reverse | GCGAGCACAGAATTAATACGAC |
| **Primers for HBXIP methylation analysis** | | |
| BSP | forward | TAAGTATTGTTTGGTGAAATGGTA |
|  | reverse | AAAATAATCTCAATCTCCTAACCTC |
| MSP | forward | AATAGGGTAGGGTGTAGTGGTTTAC |
|  | reverse | TTTTTAATAAAAATAAAATTTCGCC |
| **Primers for HBXIP promoter cloning** | | |
| p(-3233/-1673) | forward | GGTACCCAGGCGTAAACCACCATA |
|  | reverse | AAGCTTACAGCCTACCTTCCAATC |
| p(-1484/+1) | forward | GGTACCGGGTGAGGCTTACCAATT |
|  | reverse | AAGCTTCAGTGCTTCAGTTCGTGGC |
| p(-804/+1) | forward | GGTACCTTTTGGGAGGCTGGAGTG |
|  | reverse | AAGCTTCAGTGCTTCAGTTCGTGGC |
| p(-588/+1) | forward | GGTACCACAGCCTGGGTAACAGAGCA |
|  | reverse | AAGCTTCAGTGCTTCAGTTCGTGGC |
| p(-168/+1) | forward | GGTACCGGTCCCGTGTCTTC |
|  | reverse | AAGCTTCAGTGCTTCAGTTCGTGGC |
| **Primers for miR-520b promoter** | | |
| Wide type | forward | CCGGAATTCAAACAGAACCCCACCATCA |
|  | reverse | CCGCTCGAGAACAGGGCAAATAAATGCA |
| Sp1 Mut | forward | AGAGGTATGAGCCACATGTGTCAACCTGTACTTGTGTG |
|  | reverse | CACACAAGTACAGGTTGACACATGTGGCTCATACCTCT |

**Additional file 2: Table S2. MicroRNAs are regulated in LO2-X-S cells**

| miRNA | **t* test score | Fold change | Up- or down- regulation |
| --- | --- | --- | --- |
| hsa-let-7a | 25.41480993 | 3.857223001 | ↑ |
| hsa-miR-29a^#^ | 24.97052068 | 5.312362246 | ↑ |
| hsa-miR-29b | 22.63840371 | 3.475598455 | ↑ |
| hsa-miR-181c^#^ | 17.45534691 | 7.096949705 | ↑ |
| hsa-let-7i | 16.55372476 | 3.488008925 | ↑ |
| hsa-miR-422b | 12.72674147 | 3.124354232 | ↑ |
| hsa-miR-638 | 12.50664545 | 3.919626704 | ↑ |
| rno-miR-151* | 12.12611217 | 3.537590939 | ↑ |
| hsa-let-7f | 11.64965252 | 3.1922734 | ↑ |
| hsa-miR-100 | 11.47157138 | 3.793981216 | ↑ |
| hsa-miR-181d | 11.32691036 | 4.379841304 | ↑ |
| hsa-miR-181a | 9.225302371 | 3.00913355 | ↑ |
| rno-miR-31 | 8.333407321 | 3.560015306 | ↑ |
| PREDICTED_MIR189 | 7.197774926 | 8.6946 | ↑ |
| hsa-miR-193a | -30.96423431 | 0.11518621 | ↓ |
| hsa-miR-338 | -22.17468382 | 0.073524676 | ↓ |
| rno-miR-347 | -16.32194037 | 0.207016924 | ↓ |
| hsa-miR-155 | -15.85648797 | 0.183337906 | ↓ |
| PREDICTED_MIR90 | -14.56057297 | 0.240610096 | ↓ |
| hsa-miR-32 | -14.24498586 | 0.305994311 | ↓ |
| hsa-miR-365 | -14.06836533 | 0.297247766 | ↓ |
| hsa-miR-496 | -11.67960266 | 0.198111833 | ↓ |
| hsa-miR-523 | -10.78594338 | 0.289121096 | ↓ |
| hsa-miR-582 | -10.70899833 | 0.231101957 | ↓ |
| hsa-miR-520e^#^ | -10.19077967 | 0.285321128 | ↓ |
| rno-miR-20a* | -9.431989464 | 0.251538186 | ↓ |
| PREDICTED_MIR154 | -9.105426752 | 0.311688444 | ↓ |
| mmu-miR-322 | -8.988213319 | 0.256678831 | ↓ |
| hsa-miR-139 | -8.86032508 | 0.323912703 | ↓ |
| hsa-miR-30e-5p | -7.74801121 | 0.317526929 | ↓ |
| hsa-miR-520b^#^ | -6.508715059 | 0.317537502 | ↓ |
| rno-miR-224 | -5.317641321 | 0.4077 | ↓ |
| hsa-miR-30a | -4.82872289 | 0.4273 | ↓ |

All differentially expressed miRNAs have a q value<0.01(false-positive rate).

* P<0.05, Student’s *t* test. MiRNAs marked # were further tested by qRT-PCR.
